# Engagement of public and private medical facilities in tuberculosis care in Myanmar: contributions and trends over an eight-year period

**DOI:** 10.1186/s40249-017-0337-8

**Published:** 2017-09-01

**Authors:** Thin Thin Nwe, Saw Saw, Le Le Win, Myo Myo Mon, Johan van Griensven, Shuisen Zhou, Palanivel Chinnakali, Safieh Shah, Saw Thein, Si Thu Aung

**Affiliations:** 1 Procurement Unit, Department of Public Health, Naypyitaw, Myanmar; 2 Department of Medical Research, Yangon, Myanmar; 3 Institute of Tropical Medicine, Antwerp, Belgium; 4 Institute of Parasitic Diseases, Chinese Center for Disease Control and Prevention, Shanghai, China; 5 Jawaharlal Institute of Postgraduate Medical Education and Research (JIPMER), Puducherry, India; 6 Operational Research Unit (LuxOR), Médecins Sans Frontières – Operational Centre Brussels, Luxembourg, Luxembourg; 7 National TB Program, Department of Public Health, Naypyitaw, Myanmar; 8 Procurement and Supply Division, Department of Public Health, Ministry of Health, Naypyitaw, Myanmar

**Keywords:** Public and private, Tuberculosis, Myanmar, Operational research

## Abstract

**Background:**

As part of the WHO End TB strategy, national tuberculosis (TB) programs increasingly aim to engage all private and public TB care providers. Engagement of communities, civil society organizations and public and private care provider is the second pillar of the End TB strategy. In Myanmar, this entails the public-public and public-private mix (PPM) approach. The public-public mix refers to public hospital TB services, with reporting to the national TB program (NTP). The public-private mix refers to private general practitioners providing TB services including TB diagnosis, treatment and reporting to NTP. The aim of this study was to assess whether PPM activities can be scaled-up nationally and can be sustained over time.

**Methods:**

Using 2007–2014 aggregated program data, we collected information from NTP and non-NTP actors on 1) the number of TB cases detected and their relative contribution to the national case load; 2) the type of TB cases detected; 3) their treatment outcomes.

**Results:**

The total number of TB cases detected per year nationally increased from 133,547 in 2007 to 142,587 in 2014. The contribution of private practitioners increased from 11% in 2007 to 18% in 2014, and from 1.8% to 4.6% for public hospitals. The NTP contribution decreased from 87% in 2007 to 77% in 2014. A similar pattern was seen in the number of new smear (+) TB cases (31% of all TB cases) and retreatment cases, which represented 7.8% of all TB cases. For new smear (+) TB cases, adverse outcomes were more common in public hospitals, with more patients dying, lost to follow up or not having their treatment outcome evaluated. Patients treated by private practitioners were more frequently lost to follow up (8%). Adverse treatment outcomes in retreatment cases were particularly common (59%) in public hospitals for various reasons, predominantly due to patients dying (26%) or not being evaluated (10%). In private clinics, treatment failure tended to be more common (8%).

**Conclusions:**

The contribution of non-NTP actors to TB detection at the national level increased over time, with the largest contribution by private practitioners involved in PPM. Treatment outcomes were fair. Our findings confirm the role of PPM in national TB programs. To achieve the End TB targets, further expansion of PPM to engage all public and private medical facilities should be targeted.

**Electronic supplementary material:**

The online version of this article (doi:10.1186/s40249-017-0337-8) contains supplementary material, which is available to authorized users.

## Multilingual abstracts

Please see Additional file 1 for translations of the abstract into the five official working languages of the United Nations.

## Background

Tuberculosis (TB) remains one of the major global health problems, with a total 9.6 million of TB cases and 1.6 million of TB deaths reported in 2014. In response to this, the international community has engaged in ambitious global initiatives. In 2015, the World Health Organization (WHO) launched the End TB strategy, which aims to reduce TB deaths by 95% and new cases by 90% between 2015 and 2035. The strategy comprises of three pillars with a total of ten components, including enhanced case detection and case holding, engagement of all public and private TB providers, and operational research to assess progress and identify barriers and gaps [1]. Engagement of communities, civil society organizations and public and private care provider is the second pillar of the End TB Strategy [2]. Myanmar is listed among the 30 countries with a high burden of TB, TB/HIV and multidrug-resistant (MDR) TB [3]. The estimated incidence of all TB cases was 369/100000 populations/year and prevalence was 457/100000. The estimated TB mortality was 53/100000. Notification for all forms of TB cases was 293/100000 population and for bacteriologically confirmed was 293/100000 populations [4]. The National TB program (NTP) leads the TB control activities in Myanmar, collaborating with nongovernmental organization (NGO) and international NGOs and volunteers according to the National Health Plan (NHP) in line with the WHO stop TB strategy and End TB strategy. In Myanmar, sick persons use general practitioners (GPs) as their first access point for care, as these are easily accessible. They will turn to the hospital, if not improving after care by the GP, or if financial means have been exhausted.

In Myanmar, the engagement of non-NTP actors was formalized as the public-public and public-private mix (PPM) approach. PPM Directly Observed Treatment Short Course (DOTS) implies inclusion of all public and private health care providers not yet involved in DOTS implementation. Prior to this, TB care was provided by these actors outside of the NTP. Since the activities of these non-NTP actors, public and private hospitals and private general practitioners were not notified to the NTP, it was unclear to what extent national guidelines were followed, and the national TB burden was underestimated. The PPM initiative aims to strengthen the link between the NTP, the public hospitals and the private actors within the framework of the DOTS strategy. The public-public mix entails public hospitals providing diagnostic, and possibly also treatment services, with reporting to the NTP. The public-private mix refers to NGOs and private general practitioners providing these TB services.

Although engagement of all TB care providers in national TB care is one of the key pillars of the End TB strategy implemented in many low income and high burden countries in Asia, studies from the region have reported heterogeneous and sometimes conflicting findings. While involving private actors successfully increased TB case detection in most studies [5], some studies reported good treatment outcomes while others found high rates of unfavorable outcomes [6]. Moreover, the available data mainly originate from pilot projects, projects conducted in geographically limited areas (eg. one district), and/or studies covering a short period [7]. It is thus not clear whether the documented contributions are sustained over time, or whether these can be realized at the national level. Data looking at the integration of non-NTP public actors are even scarcer. What is missing are evaluations, at the national level and over a long-term period, of the activities and contributions of all non-NTP providers to TB case detection and treatment. This is an important gap, as TB elimination is unlikely to be achieved without effective partnerships between all TB care providers, supported by operational research evaluating the joined efforts and identifying shared or specific program challenges.

Using routine program data collected by the NTP from public-public and public-private actors between 2007 and 2014, we analyze 1) the number of TB cases diagnosed and their relative contribution to the national case load; 2) the demographic characteristics and type of TB cases diagnosed; 3) their treatment outcomes.

## Methods

### Study design

A retrospective analysis using routinely collected data.

### Study setting

Myanmar is a South-East Asian country with a total population of 51.4 million. Administratively, the country is divided into seven regions and seven states. Each region or state consists of a number of districts (74 in total), which are further divided into townships (330 in total). In 2015, WHO estimated that the TB incidence was 369 per 100,000 population, TB prevalence was 457 per 100,000 population, and that the estimated incidence of new smear-positive cases was 105 per 100,000 population. The NTP falls under the Department of Public Health of the Ministry of Health and Sports (MOHS). The NTP is currently running 17 Regional and State TB Centers including Naypyitaw, capital city of Myanmar with 101 TB teams at district and township levels. All 330 townships in Myanmar currently function according to the DOTS strategy. It is the internationally recommended strategy for TB control that has been recognized as a highly efficient and cost-effective. DOTS comprises five components: (1) sustained political and financial commitment, (2) diagnosis by quality ensured sputum-smear microscopy, (3) standardized short-course anti-TB treatment (SCC) given under direct and supportive observation, (4) a regular, uninterrupted supply of high quality anti-TB drugs, and (5) standardized recording and reporting [8]. Public hospitals fall under the Department of Medical Care of the MOH and have traditionally – before PPM - provided TB care without direct link to the NTP. The private health care sector plays and increasingly prominent role in health care provision in Myanmar. While most private hospitals can be found in larger cities, private general practitioners are located throughout the country and are mainly engaged in ambulatory care. GPs are medical doctors trained by the medical university. More than 3000 medical doctors have been trained by medical universities.

### The public-private mix (PPM) strategy

#### The public-public mix

This collaboration aims to strengthen the link between the NTP and the public hospitals within the framework of the DOTS strategy. This started with an advocacy meeting, following by a 2 days training of the hospital staff. A hospital DOTS committee was formed and chaired by the medical superintendent and the heads of the clinical departments. A specific DOTS corner was formed, for TB case detection, drug supply and recording and reporting. A TB focal person was assigned, supervised by the Assistant Medical superintendent (AMS).

In 2007, linkage of TB activities in these public hospitals to NTP was piloted in six hospitals, with gradual scaling-up since then. Currently, 24 hospitals are involved. While originally four options with different degrees of involvement were proposed, all PPM hospitals are currently implementing option 3 and option 4. Option 3 refers to patients diagnosed and started on TB treatment in the hospital followed by referral to the NTP health center during treatment. Under option 4, patients are diagnosed, treated and have their outcome ascertained in the hospital, with reporting to the NTP. Option 1 involves diagnosis of TB cases, prescription of TB treatment in hospital, followed by referral to township for DOT, with clinical follow-up at the hospital. Option 2 involves diagnosis of TB cases, prescription of TB treatment in hospital, followed by referral to township for DOT, without clinical follow-up at the hospital.

#### The public-private mix

The public-private mix entails the involvement of national and international NGOs and private practitioners in TB services including TB case finding, case holding and reporting to NTP.

The first initiatives date back to 2004. Currently a total of one national and six international NGOs are involved in the public-private mix. Most international NGOs provide TB care as part of their direct program activities, for example integrated in HIV care programs.

The engagement of private practitioners in TB care is mainly organized via one international (Population Services International - PSI) and one national NGO (Myanmar Medical Association - MMA).

Most private general practitioners have their own private clinic, few are organized in poly-clinics or special clinics. For sputum examination, private doctors refer TB suspects to NTP labs and PSI or MMA affiliated private labs, accredited by the NTP and monitored under an external quality assurance system (EQA). The key contributions of the NGOs are provision of TB drugs – provided by the NTP – and support in reporting to NTP on the TB activities. Supervision is conducted jointly by the NTP, WHO and the respective NGO.

Private practitioners can engage into TB care according to three schemes, with increasing involvement. Scheme 1 consists of health education and referral of presumptive TB cases. In Scheme 2, they additionally function as DOT providers. Scheme 3 refers to NTP affiliated DOTS centers/clinics, which often have an NTP accredited private laboratory.

### Study participants

All TB cases diagnosed between 2007 and 2014 by the public-public mix according to option 4, the public-private mix and the NTP in Myanmar. The focus on option 4 for the public-public mix is because these patients are entirely managed outside of the NTP and reported separately. Information on patients managed under option 3 are not reported separately, but – as they are referred for treatment to the NTP - feature in the aggregated NTP report.

### Source of data, data collection and validation

All TB actors (all services providing TB services including the public-public and public-private mix) in Myanmar have to report aggregated program data to the NTP on a quarterly basis using a standard reporting form developed by the NTP. This information is entered by the NTP in an Excel-based database, from which the data were sourced for this study. All sites involved in TB care are at least yearly visited by the NTP to check on completeness and validity of the reported data. The following aggregated data were extracted per each TB actor and for each year: the number of TB cases diagnosed, demographics (age, sex), and type of TB and treatment outcomes. TB cases were classified as new or retreatment. New cases were further stratified as smear (+) pulmonary TB, smear (−) pulmonary TB and extra-pulmonary TB (EPTB). In 2014, this was modified as bacteriologically confirmed PTB, clinically diagnosed PTB, bacteriologically confirmed EPTB, clinically diagnosed EPTB. Treatment outcomes were classified as cured, treatment completed, died, failure, loss to follow-up, or outcome not evaluated (definitions provided in the Table 1).

**Table 1:** Myanmar’s national TB treatment guideline definition for TB treatment outcome

Outcomes	Definitions
Cure	A pulmonary TB patient with bacteriologically confirmed TB at the beginning of treatment who was smear or culture negative in the last month of treatment and on at least one previous occasion.
Treatment completed	A TB patient who completed treatment without evidence of failure but with no record to show that sputum smear or culture results in the last month of treatment and on at least one previous occasion were negative, either because tests were not done or because results are unavailable.
Treatment failure	A TB patient whose sputum smear or culture is positive at month 5 or later during treatment.
Died	A TB patient who dies for any reason before starting or during the course of treatment.
Loss to follow-up	A TB patient who did not start treatment or whose treatment was interrupted for 2 consecutive months or more.
Not evaluated	A “Transferred out” patient whose treatment outcome is not received (unknown) from the transferred township.

### Analysis and statistics

Data on the TB burden, patient’s characteristics and treatment outcomes were summarized using frequencies and percentages. Trends over time were being graphically represented.

## Results

**Fig. 1:**
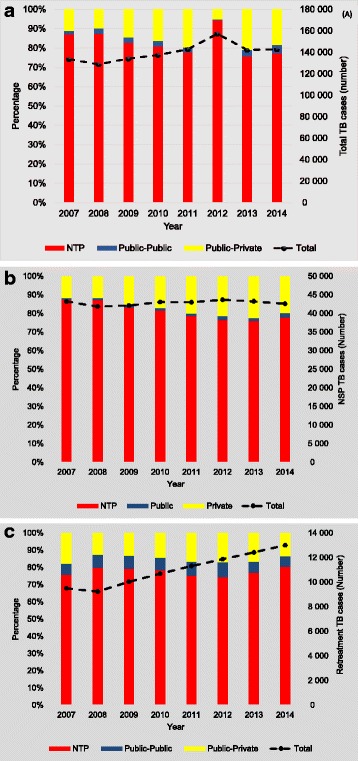
Tuberculosis (TB) cases diagnosed and relative contribution of National TB program, public-public and public-private partnerships in Myanmar, 2007–2014. **a** TB case detection (all forms). **b** New smear(+) TB case detection. **c** Retreatment TB case detection

The total number of cases detected per year ranged from 133,547 in 2007, to 137,403 in 2010 and 142,587 in 2014, with a peak in 2012 (Fig. 1a). The contribution of private practitioners increased from 11% in 2007 to 18% in 2014, and from 1.8% to 4.6% for public hospitals. The contribution of NTP in national TB case detection decreased from 87% in 2007 to 77% in 2014.

A similar pattern for public hospitals and private practitioners was seen in the number of new smear(+) TB cases (Fig. 1b) and retreatment cases (Fig. 1c), with as main difference that the absolute numbers of new smear(+) TB cases detected by NTP decreased over time, but the numbers of retreatment cases increased. The contribution of new smear positive TB cases for NTP decreased from 87% in 2007 to 78% in 2014, but increased for private practitioners from 12% to 20% and for public hospitals from 1% to 2% in 2007 and 2014, respectively. The contribution of retreatment TB cases for NTP increased from 76% in 2007 to 81% in 2014, but decreased for private practitioners from 18% to 13% and for public hospitals from 6% to 6% in 2007 and 2014, respectively. The demographics of the new smear (+) TB cases are shown in Table 2. Most (65%) were male and between 25 to 44 years old. No clear differences were noted between the three different providers.

**Table 2:** Age and gender distribution of sputum smear (+) tuberculosis (TB) cases diagnosed by National TB program (NTP), public-public and public-private partnerships in Myanmar, 2007–2014

	NTP		Private		Public (non-NTP)	
	*N*	%	*N*	%	*N*	%
Total	339,989		74,959		9672	
Sex						
Male	220,360	65	42,118	64	6300	65
Female	119,629	35	26,481	36	3372	35
Age (years)						
0–14	29,620	9	7361	10	1361	14
15–24	41,227	12	9821	13	863	9
25–34	67,295	20	16,769	22	2741	28
35–44	67,795	20	14,925	20	2329	24
45–54	59,898	17	12,088	16	1240	13
55–64	43,201	13	8297	11	658	7
≥ 65	30,953	9	5693	18	480	5

*NTP* national tuberculosis program

**Fig. 2:**
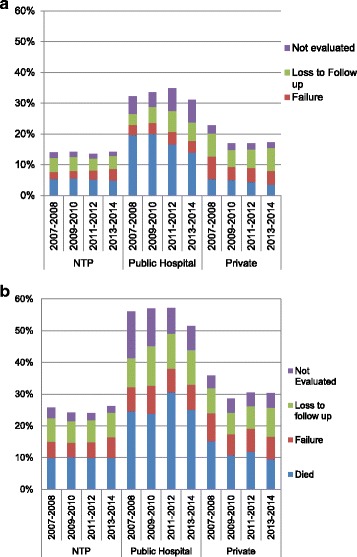
Unfavorable Tuberculosis (TB) treatment outcomes of new smear positive TB cases by type of provider in Myanmar, 2007–2014. **a** new smear positive TB cases. **b** retreatment TB cases

The treatment outcomes are presented in Fig. 2. For new smear(+) TB cases, adverse outcomes were more common in public hospitals, mainly due to an increased proportion of patients that died, were lost to follow up or were not evaluated (Fig. 2a). Patients treated by private practitioners were more frequently loss to follow up. Adverse treatment outcomes in retreatment cases were particularly common in public hospitals for various reasons, predominantly due to patients dying or not having their treatment outcome evaluated (Fig. 2b). In private clinics, treatment failure tended to be more common.

## Discussion

Over the eight year period, the contribution of non-NTP actors to TB detection at the national level increased from 13% in 2007 to 23% in 2014, with the largest contribution by private practitioners involved in PPM. Treatment outcomes by private practitioners were generally good, although the reasons behind the higher default rate in the private sector remain unclear. Treatment outcomes were less satisfactory in the public hospital, particularly for retreatment cases.

Our findings of the successful contribution of PPM to TB case detection are in line with previous studies from Myanmar and neighboring countries [5, 9, 10]. Our study adds to the existing literature by providing information on what PPM can represent at the national level, and by demonstrating that this can be sustained, and scaled-up, over a long period. For instance, a pilot project in Myanmar conducted between 2002 and 2006 showed encouraging results, but emphasized that sustaining and replicating such partnerships might be challenging [7], and similar concerns have been expressed in other studies from the region [11, 12].

Besides a peak in 2012, the total case detection tended to increase slightly between 2007 and 2014. The increase in 2012 can be explained by a number of specific activities resulting in exceptional high detection rates by the NTP, including the launch of a TB-reach active case finding project [13].

The public-private mix made a substantial contribution to TB case detection. Qualitative studies in Myanmar from 10 to 15 years ago identified multiple gaps and limited collaboration between the NTP and private general practitioners [14, 15] Limited trust between public and private sector and lack of knowledge on national TB guidelines amongst general practitioners were put forwards as contributing factors [14]. The current success indicates that some of these have been overcome. Key factors for successful and sustainable PPM-DOTS that have previously been identified include advocacy and increased awareness raising, regular continued medical education (CME) activities, good coordination of PPM and regular contact between all actors [16].

However, there is also room for improvement. With the vast majority of TB patients seeking care from the private sector before presenting at the TB center, partnership with the entire private sector is needed for success towards TB elimination. It is estimated that around 40–45% of the general practitioners currently still provide TB care outside PPM, prescribing TB drugs via drug shops, and without notification to the NTP [17]. Factors previously put forward by non-PPM general practitioners for their non-participation included the paperwork required for PPM and insufficient recognition of their contribution by the NTP [16].

These factors have been addressed by the NTPS since then. It is also encouraging that almost all GPs are member of the Myanmar medical association, providing opportunities for further awareness raising and increasing partnerships. As the most recent qualitative study on PPM dates back from 2009, more up to date information on potential barriers and success factors would be useful. Finally, increased access of diagnostic innovations such as Gene X pert for non-NTP actors could further enhance case detection.

While previous studies from pilot projects have reported good treatment outcomes within the public private mix [17], the default rate in our study was relatively high. Whether there are other contributing factors such the quality of DOTS, patient support and tracing requires further in-depth assessment.

The contribution of the public-public mix remained small, with only 24 tertiary level hospitals involved. However, as information on the activities of the public hospitals referring TB cases for treatment to the NTP – I e working under option 3 – was not available, their contribution is underestimated in our study. The next step would be the integration of the district hospitals, which currently provide TB care as part of the primary health care program, under a different department. Specialty hospitals with a high TB burden such as specialist hospitals should be preferentially targeted as well, besides hospitals falling under other ministries than the MOHS (eg. military and railway hospitals). Strengthened partnership with all public actors would further help to ensure completeness of reporting of national TB case detection, and alignment of TB care with national TB guidelines. The higher mortality rate in TB cases in the public hospitals, compared to the NTP, is likely explained by disease stage or co-morbidities. Follow-up studies would be of value to assess opportunities for further reducing the case fatality rate. The reasons for the high proportion of default and patients without outcome evaluation, particularly amongst retreatment cases, should be determined as well.

One of the strengths of this study is the use of national data, representative of what is happening in the country. Data validation systems were in place across the eight year period. In contrast with most other studies, our study also covered a long period, allowing assessing the long-term contribution of PPM. It is also one of the few studies concurrently evaluating public-public and public-private partnerships. There are a number of important limitations to acknowledge. The use of aggregated data program data precluded more in depth analysis. As per NTP reporting guidelines, demographic information was only available for new smear (+) TB cases.

## Conclusions

In conclusion, between 2007 and 2014, the PPM model contributed substantially and increasingly to TB case detection. While the contribution of the public-private mix reached 18% in 2014, this remained at 4.5% for the public-public mix. Treatment outcomes were generally fair, although the higher loss to follow up rate in the private sector and the overall poorer outcomes in retreatment cases in public hospitals needs attention. Further scaling-up of the PPM model will require finding ways to engage the relatively large group of general practitioners not yet involved in PPM, and integration of all hospitals in TB care.

## Additional file

Additional file 1: Multilingual abstracts in the five official working languages of the United Nations. (PDF 680 kb)

## Abbreviations

DOTS: Directly Observed Treatment Short Course
EPTB: Extra Pulmonary Tuberculosis
EQA: External Quality Assurance
GPs: General Practitioners
MMA: Myanmar Medical Association
MOHS: Ministry of Health and Sport
NGOs: Nongovernmental Organizations
NTP: National Tuberculosis Program
PPM: Public-Public-Public-Private-Mixed
PSI: Populations Services International
PTB: Pulmonary Tuberculosis
TB: Tuberculosis
WHO: World Health Organization
