# Supplementary material for: Engagement of public and private medical facilities in tuberculosis care in Myanmar: contributions and trends over an eight-year period
Source: Infect Dis Poverty. 2017 Sep 1;6:123. doi: 10.1186/s40249-017-0337-8 (PMC5579949; doi:10.1186/s40249-017-0337-8)
Supplement: Additional file 1: — Multilingual abstracts in the five official working languages of the United Nations. (PDF 680 kb) [file 40249_2017_337_MOESM1_ESM.pdf]

Translation of the abstract into the five official working languages of the United Nations

إشراك المرافق الطبية العامة والخاصة في مجال رعاية السل في ميانمار: المساهمات والاتجاهات خلال فترة 8 سنوات

ثين ثين نيو، ساو ساو، لو لي وين، ميو ميو مون، يوهان فان جرينسفين، شويسن تشو، بالانيفل تشيناكالي، صفية شاه، ساو ثين، سي ثو أونج

ملخص

خلفية: كجزء من استراتيجية منظمة الصحة العالمية لمكافحة السل، تهدف البرامج الوطنية لمكافحة السل بصورة متزايدة إلى إشراك جميع مقدمي الرعاية الخاصة والعامة في مجال السل. ويشكل إشراك المجتمعات المحلية ومنظمات المجتمع المدني ومقدمي الرعاية من القطاعين العام والخاص الركيزة الثانية لاستراتيجية القضاء على السل. وفي ميانمار، يستتبع هذا النهج المتبع بين القطاعين العام والخاص والخاص بين القطاعين العام والخاص. ويشير امتزاج العمل العام إلى الخدمات المقدة لمرضى السل في المستشفيات العامة، مع تقديم التقارير إلى البرنامج الوطني لمكافحة السل. وامتزاج العمل بين القطاعين العام والخاص يشير إلى قيام الممارسين العامين في القطاع الخاص بتوفير خدمات المقدمة لمرضى السل، بما في ذلك تشخيص مرض السل، والعلاج، وتقديم التقارير إلى البرنامج الوطني لمكافحة السل. وكان الهدف من هذه الدراسة لتقييم ما إذا كان يمكن أن تكون زيادة في التعاون البيني بين الجهات العامة والتعاون بين الجهات العامة والخاصة، وإذا كان يمكن أن يستمر.

الأساليب: استخدام بيانات البرنامج المجمعة في الفترة 2007-2014، جمعنا المعلومات من الجهات الفاعلة في البرنامج الوطني لمكافحة السل والجهات الأخرى 1) عدد حالات السل التي اكتشفت ومساهمتها النسبية في عدد الحالات على المستوى الوطني؛ 2) نوع حالات السل المكتشفة؛ 3) نتائج العلاج.

النتائج: العدد الإجمالي لحالات السل التي اكتشفت في السنة على الصعيد الوطني زاد من 133 547 في عام 2007 إلى 142 587 في عام 2014. مساهمة الممارسين في القطاع الخاص زادت من 11 في المائة في عام 2007 إلى 18 في المائة في عام 2014، ومن 1.8 في المائة إلى 4.6 في المائة للمستشفيات العامة. مساهمة البرنامج الوطني لمكافحة السل انخفضت من 87 في المائة في عام 2007 إلى 77 في المائة في عام 2014. وكان ينظر إلى نمط مماثل في عدد حالات السل الإيجابية اللُّطَاخة الجديدة (31 في المائة من جميع حالات الإصابة بالسل) وحالات إعادة المعالجة، الذي يمثل 7.8 في المائة من جميع حالات الإصابة بالسل. لحالات السل الإيجابية اللُّطَاخة الجديدة، النتائج السلبية كانت أكثر شيوعاً في المستشفيات العامة، مع زيادة في المرضى المتوفين، أو لم يتم متابعة حالاتهم أو لم يتم تقييم نتائج العلاج الخاصة بهم. كان عدد المرضى الذين لم يتم متابعةعلاجهم بو اسطة المما رسين في القطاع الخاص أشيع (8% في المائة). كانت نتائج العلاج سلبية في حالات إعادة المعالجة شائعة بوجه خاص (59 في المائة) في المستشفيات العامة لأسباب مختلفة، غالباً بسبب وفاة المرضى (26 في المائة) أوعدم تقييم العلاج (10%). في العيادات الخاصة، زادت نسبة الفشل في العلاج (8%).

الاستنتاج: <bx/>زاد إسهام الجهات الفاعلة غير التابعة للبرنامج القومي لمكافحة السل في الكشف عن مرض السل على الصعيد الوطني، مع مساهمة أكبر من الممارسين في القطاع الخاص في العمل المشترك بين مقدمي الرعاية الخاصة والعامة في مكافحة السل. كانت نتائج المعالجة مقبولة. وتؤكد النتائج التي توصلنا إليها دور العمل المشترك بين مقدمي الرعاية الخاصة والعامة في مكافحة السل في البرامج الوطنية لمرض السل. لتحقيق أهداف القضاء على السل، يجب توسيع إشراك جميع المرافق الطبية العامة والخاصة في مكافحة السل.

Translated from English version into Arabic by Mahmoud Sami, through

TRANSLATORS WITHOUT BORDERS

缅甸的公共和私人医疗机构在 8 年间参与结核病治疗所做的贡献及发展趋势

Thin Thin New, Saw Saw, Le Le Win, Myo Myo Mon, Johan van Griensven, Shuisen Zhou, Palanivel Chinnakali, Safieh Shah, Saw Thein, Si Thu Aung

摘要

引言：作为世界卫生组织消除结核病战略的一部分，缅甸国家结核病防治计划（NTP）逐渐致力于吸引所有私人和公共的结核病医疗提供者。社区、民间社会组织、公共和私人医疗提供者的参与是消除结核病战略的第二支柱。在缅甸就是公共-公共混合和公私混合（PPM）医疗两种参与方式。公共-公共混合是指公立医院的结核病医疗服务与 NTP 报告相结合。公私混合是指提供结核病服务的私人全科医生所提供的服务包括结核病诊断、治疗和向国家结核病规划项目报告。本研究的目的在于评估 PPM 这种方式是否能在全国范围内展开，并持续下去。
方法：使用 2007-2014 年汇总的项目数据，分别从 NTP 和非 NTP 参与者处收集以下几方面的信息：1）发现的结核病例数及其对国家病例数的相关贡献，2）发现的结核病例类型，3）治疗结果。
结果：缅甸全国每年检出的结核病例数从 2007 年的 133 547 例增至 2014 年的 142 587 例。私人执业医生的贡献由 2007 年的 11%增至 2014 年的 18%，公立医院的贡献由 1.8%增至 4.6%。NTP 的贡献从 2007 年的 87%下降到 2014 年的 77%。2007-2014 年间，私人执业医生和公立医院在新涂阳结核病例（占总病例数的 31%）和复治病例（占 7.8%）诊治方面的贡献情况类似。对于新涂阳结核病例，在公立医院中，不良治疗结果更为常见，死亡病例、失访患者或未评估治疗结果的患者更多；而私人医生治疗的患者多失访（8%）。在公立医院由于各种原因，复治病例的不良治疗结果尤为普遍（59%），主要包括患者死亡（26%）或未评估治疗结果（10%）。在私人诊所，治疗失败往往更为常见（8%）。
结论：在全国范围内，非 NTP 参与者对结核病检测的贡献随着时间的推移而增加，参与 PPM 的私人执业医师贡献最大，治疗结果合理。我们的研究结果证实了 PPM 在国家结核病防治计划中的作用。为了实现消除结核病的目标，需进一步扩大 PPM 以吸引所有公私人医疗机构参与进来。

Translated from English version into Chinese by Zhou Guan, edited by Pin Yang

CHINESE CENTER FOR DISEASE CONTROL AND PREVENTION
NATIONAL INSTITUTE OF PARASITIC DISEASES

Implication des établissements médicaux publics et privés dans les soins de la tuberculose au Myanmar: contributions et tendances sur une période de 8 ans

Thin Thin New, Saw Saw, Le Le Win, Myo Myo Mon, Johan van Griensven, Shuisen Zhou, Palanivel Chinnakali, Safieh Shah, Saw Thein, Si Thu Aung

RÉSUMÉS

Rappel des faits: Dans le cadre de la stratégie d'éradication de la tuberculose, les programmes nationaux de lutte contre la tuberculose (TB) visent de plus en plus à impliquer tous les personnels de santé privés et publics. Au Myanmar, cela implique l'approche mixte du public-public et du public-privé (PPM). Le mélange public-public se réfère aux services de tuberculose des hôpitaux publics, qui rendent des comptes au programme national de lutte contre la tuberculose (PNT). Le mélange public-privé fait référence aux médecins généralistes privés qui offrent des services de lutte contre la tuberculose. Alors que les petits projets ont fourni des résultats encourageants à court terme sur l'approche PPM, il est encore difficile de savoir si cela peut être introduit au niveau national et maintenu dans la durée.
Méthode: En utilisant les données agrégées du programme 2007-2014, nous avons recueilli des informations auprès des acteurs du PNT et ceux n'appartenant pas au PNT sur : 1) le nombre de cas de tuberculose diagnostiqués et leur impact relatif au nombre de cas à l'échelle nationale; 2) le type de cas de tuberculose diagnostiqués; 3) les résultats de leur traitement.
Résultats: Le nombre total de cas de tuberculose détectés par an a augmenté de 133,547 en 2007 à 142,587 en 2014. La contribution des praticiens privés est passée de 11% en 2007 à 18% en 2014 et de 1,8% à 4,6% pour les hôpitaux publics.

La contribution du PNT est passée de 87% en 2007 à 77% en 2014. Un taux similaire a été observé pour le nombre de nouveaux cas de tuberculose (31% de tous les cas de tuberculose) et de cas de re-traitement, qui représentaient 7,8% de tous les cas de tuberculose. Pour les nouveaux cas de tuberculose, les résultats défavorables étaient plus fréquents dans les hôpitaux publics, avec plus de décès de patients, un manque de suivi ou un manque d'évaluation des résultats du traitement. Les patients pris en charge par des médecins privés souffrent souvent d'un manque de suivi. Les effets indésirables du traitement dans les cas de re-traitement sont particulièrement fréquents dans les hôpitaux publics pour diverses raisons, principalement en raison du décès ou de l'absence d'évaluation des patients. Dans les cliniques privées, l'échec du traitement est aussi plus fréquent.

Conclusion: La contribution des acteurs n'appartenant pas au PNT dans la détection de la tuberculose au niveau national a augmenté au fil du temps, avec un grand nombre de praticiens privés impliqués dans le PPM. Pour atteindre les objectifs du programme de lutte contre la tuberculose, il est important de viser une extension supplémentaire du PPM, afin d'engager toutes les infrastructures médicales publiques et privées.

Translated from English version into French by Debizetrad, through

TRANSLATORS
WITHOUT BORDERS

Участие государственных и частных медицинских учреждений Мьянмы в лечении туберкулеза: вклад и тенденции за 8-летий период

Тин Тин Нью, Соу Соу, Ли Ли Уин, Мьё Мьё Мон, Йохан ван Гриенсвен, Шуйзен Чжоу, Паланивель Чиннакали, Сафий Шах, Соу Тейн, Си Ту Аунь

РЕФЕРАТ

Справочная информация: В рамках стратегии ВОЗ по ликвидации туберкулёза (ТБ), национальные программы по борьбе с указанным заболеваниям всё более нацелены на задействие как частных, так и государственных работников здравоохранения, специализирующихся на лечении ТБ. Вовлечение сообществ, организаций гражданского общества, а также государственных и частных медицинских учреждений является вторым ключевым элементом в стратегии по ликвидации ТБ. В случае Мьянмы это означает как государственный подход, так и смешанный (далее именуемый PPM), задействующий как частный, так и государственный секторы. Под государственным подходом подразумеваются услуги по лечению туберкулёза, предоставляемые государственными больницами, с последующей передачей отчетов в национальные программы по борьбе с ТБ (далее именуемые NTP). Цель данного научного исследования заключалась в оценке возможности расширения деятельности PPM на национальном уровне с сохранением её устойчивости в долгосрочной перспективе.

Методы: Использование обобщённых програмных данных за период с 2007 по 2014 годы, собранных нами на основании информации, предоставленной как NTP, так и иными участниками, не задействованными в NTP, по следующим параметрам: 1) количество выявленных случаев ТБ и их относительное влияние на национальные нагрузки; 2) тип случаев ТБ; 3) исходы их лечения.

Результаты: Общее количество случаев заболевания туберкулёзом в год, выявленных на национальном уровне, увеличилось с 133 547 в 2007 году до 142 587 в 2014 году. Вклад частных практикующих врачей увеличился с 11% в 2007 году до 18% в 2014 году, а в государственных больницах с 1,8% до 4,6%. Вклад NTP сократился с 87% в 2007 году до 77% в 2014 году. Аналогичная картина наблюдалась в ряде новых положительных результатов мазка на туберкулез (+) (31% от всех случаев ТБ), а также при повторных случаях, составляющих 7,8% от общего числа всех заболевших ТБ. В случаях обнаружения свежих положительных результатов при мазках (+) на туберкулёз, неблагоприятные последствия чаще всего встречались в государственных больницах, на которые приходилось большинство фатальных исходов, а также недостаток в отслеживании лечения и оценки его исхода. Чаще всего неотслеженными (8%) оказывались результаты лечения, осуществляемого частными врачами. По ряду причин наиболее часто случаи нежелательного исхода при повторном лечении встречались в государственных больницах (59%), главным образом вследствие смерти пациентов (26%) или отсутствия оценки состояния здоровья (10%). В частных клиниках наиболее часто имели место случаи неэффективности лечения (8%).

Заключение: С течением времени вклад в выявление ТБ со стороны участников, не входящих в состав NTP, на национальном уровне увеличился, при содействии частных практикующих врачей, принимавших участие в PPM. Результаты лечения были добросовестными. Наши выводы подтверждают роль PPM в национальных программах по борьбе с туберкулёзом. Для достижения целей по ликвидации ТБ требуется дальнейшее расширение PPM с вовлечением всех государственных и частных медицинских учреждений.

Translated from English version into Russian by Liudmila Tomanek (nee Volynets), through

TRANSLATORS
WITHOUT BORDERS

Participación de los servicios médicos públicos y privados de atención de la tuberculosis en Myanmar: aportaciones y tendencias a lo largo de un período de 8 años

Thin Thin New, Saw Saw, Le Le Win, Myo Myo Mon, Johan van Griensven, Shuisen Zhou, Palanivel Chinnakali, Safieh Shah, Saw Thein, Si Thu Aung

RESUMEN

Introducción: Como parte de la Estrategia Alto a la TB, los programas nacionales de tuberculosis (TB) tienen como objetivo atraer a servicios médicos públicos y privados de atención de la TB. La participación de las comunidades, las organizaciones de la sociedad civil y los servicios médicos públicos y privados de atención de la tuberculosis constituyen el segundo pilar de la Estrategia Alto a la TB. En Myanmar, ello implica implementar un enfoque público-público y una alianza público-privado (APP/PPM). El enfoque público-público se refiere a los servicios contra la TB de los hospitales públicos, con la presentación de informes al programa nacional contra la TB (PNT). La alianza público-privada se refiere a las consultas privadas de medicina general que proporcionan atención contra la TB, y que incluyen diagnóstico, tratamiento e informes al PNT. El objetivo de este estudio ha sido evaluar si las actividades APP/PPM pueden ampliarse a nivel nacional y mantenerse de modo sostenido en el tiempo.

Métodos: Utilizando datos agregados programados 2007-2014, hemos recopilado información de participantes del PNT y de no participantes en el PNT sobre 1) el número de casos de TB detectados y su contribución relativa a la cantidad de casos nacionales; 2) el tipo de casos de TB detectados; 3) los resultados del tratamiento.
Resultados: El número total de casos de TB detectados por año a nivel nacional ha aumentado de 133.547 en 2007 a 142.587 en 2014. La contribución de los médicos privados ha aumentado del 11% en 2007 al 18% en 2014, y del 1,8% al 4,6% para los hospitales públicos. La contribución al PNT ha descendido del 87% en 2007 al 77% en 2014. Un patrón similar se ha observado en el número de nuevos casos de frotis (+) de TB (31% de todos los casos de TB) y casos de nuevo sometimiento al tratamiento, que representaban el 7,8% de todos los casos de TB. Para nuevos casos de frotis (+) de TB, los resultados adversos fueron más comunes en los hospitales públicos, con más fallecimientos de pacientes, que no continuaron el seguimiento o cuyos resultados del tratamiento no fueron evaluados. Ha sido mayor el número de pacientes que no continuaron el seguimiento en el caso de pacientes tratados por médicos privados (8%). Han sido especialmente frecuentes los resultados adversos al tratamiento en casos de sometimiento a un nuevo tratamiento (59%) en hospitales públicos por diversas razones, principalmente debido al fallecimiento de los pacientes (26%) o a la falta de evaluación (10%). En clínicas privadas, el fracaso del tratamiento ha tendido a ser más frecuente (8%).
Conclusión: La contribución de los no participantes en el PNT para la detección de la TB a nivel nacional ha ido aumentando en el tiempo, con la mayor contribución por parte profesionales privados involucrados en la alianza APP/PPM. Los resultados del tratamiento fueron aceptables. Nuestros hallazgos confirman el papel de la alianza APP/PPM en los programas nacionales de lucha contra la TB. Para alcanzar los objetivos de la Estrategia Alto a la TB, es necesario apuntar hacia una mayor expansión de la alianza APP/PPM para conectar todas las instalaciones médicas públicas y privadas.

Translated from English version into Spanish by Ana Claudia Macoretta, through

TRANSLATORS
WITHOUT BORDERS
